# Ebola Outbreak amid COVID-19 in the Republic of Guinea: Priorities for Achieving Control

**DOI:** 10.4269/ajtmh.21-0228

**Published:** 2021-04-14

**Authors:** Abdullahi Tunde Aborode, Christos Tsagkaris, Shubhika Jain, Shoaib Ahmad, Mohammad Yasir Essar, Emmanuel Adebowale Fajemisin, Irem Adanur, Olivier Uwishema

**Affiliations:** 1 Oli Health Magazine Organization, Research and Development, Kigali, Rwanda;; 2 Healthy African Platform, Research and Development, Ibadan, Nigeria;; 3 University of Crete, Faculty of Medicine, Heraklion, Greece;; 4 Kasturba Medical College, Manipal, Karnataka, India;; 5 Punjab Medical College, Faisalabad, Pakistan;; 6 Medical Research Center, Kateb University, Kabul, Afghanistan;; ^*7*^Department of Biochemistry, Federal University of Technology, Akure, Nigeria;; 8 SWIS Africa Platform, Research and Development, Lagos, Nigeria;; 9 Faculty of Medicine, Karadeniz Technical University, Trabzon, Turkey;; 10 Department of General Medicine, Karadeniz Technical University, Trabzon, Turkey;; 11 Oli Health Magazine Organization, Research and Development, Kigali, Rwanda

## Abstract

In February 2021, a new Ebola outbreak occurred amid the coronavirus disease 2019 (COVID-19) pandemic in the Republic of Guinea. Technical committees and Ebola mitigation mechanisms used during the 2014–2016 Ebola epidemics, have been redeployed by the public health organizations and African health organizations. As the burden on the local healthcare system is rising, fears of socioeconomic disruption are growing as well. Strategies used during the previous epidemic need to be reactivated, and new measures taken during the challenges of COVID-19 are being considered. This perspective discusses the available evidence regarding the epidemic of Ebola in Guinea amid the COVID-19 pandemic, highlights the challenges to be prioritized, and provides evidence-based recommendations.

## INTRODUCTION

In 2014, there was an epidemic of the Ebola virus in West Africa; the outbreak started in the Guinea Republic and spread from Sierra Leone to Liberia. The Ebola virus resulted in more than 11,300 deaths by June 2016, with reports of mortality reaching up to 50%.^1^ The socioeconomic burden, due to the disruption of professional activity and loss of work, was estimated to be US $53.2 billion.^2^ At that time, the reported detrimental effects of the Ebola virus disease (EVD) outbreak were attributed to multifaceted leadership mismanagement spanning from the delayed response from the WHO and from the poor coordination of national and local authorities^3,4^ to inadequate healthcare systems, limited resources and access to specialized healthcare workers, and the high level of international mobility.^5^

A new EVD outbreak, happening amid the global coronavirus disease 2019 (COVID-19) pandemic, sets the population and socioeconomic stability in peril.^6,7^ This outbreak adds to the financial instability, the closure of small and local businesses, and food shortage that have been documented during the previous months.^8^ In addition, the experience of COVID-19, along with the history of EVD, has made more urgent the need for investment in equipment, infrastructure, and personnel in an effort to better prepare healthcare facilities to treat the victims of infectious outbreaks.^9^

The Republic of Guinea government declared a resurgence Ebola on February 14, 2021, with seven reported cases from the southeastern part of Nzérékore.^9,10^ A national taskforce has been assembled to coordinate the containment of the EVD outbreak within the country. The taskforce is also working closely with countries sharing borders with Nzérékore to monitor cross-border spread of the virus. Furthermore, the WHO, Africa Central Diseases Control, and International Medical Corps are international health agencies currently combating EVD amid the COVID pandemic in Guinea Republic.^10,11^

At this point, efforts focus on informing the public, staffing the testing facilities, providing protective equipment and education on its use, maintaining an adequate level of care for other diseases, and streamlining containment and testing practices.^6,9,10^ Despite the adversity, the national response can benefit from lessons learned during the previous EVD outbreak. On these grounds, the authors discuss the response of the Republic of Guinea to EVD during the COVID-19 pandemic, elaborate on challenges and priorities, and provide evidence-based recommendations.

## IMPACT OF EBOLA VIRUS AMID THE RESPONSE TO COVID-19

Insufficient capacity to examine and isolate cases of EVD led to the growth of cases reported in the 2014–2016 outbreaks of Ebola in sub-Sahara Africa. The equipment that the country obtained at that time has been helpful for the detection of SARS-CoV-2 in the previous months.^11^ However, the current reemergence of EVD increases the burden for the available facilities and laboratories.

The state healthcare system is responsible for obtaining and transporting the samples safely, securing appropriate reagents, discarding harmful substances, and delivering the results of the tests in a timely manner. Decision-making depends on the latter. Good practices of testing for the detection of SARS-CoV-2 have been streamlined over the past months. Moreover, healthcare workers have also obtained relevant experience. Therefore, redeploying the same system for the epidemiological monitoring of the spread of Ebola is feasible, although it clearly requires additional resources and personnel.^11^

In this regard, the standard operating procedures (SOPs) serving as guidelines to Ebola response in 2014–2016 have recently been repurposed for COVID-19 SOPs. Now, Ebola and COVID-19 SOPs are redeployed in parallel, maintaining similar contact tracing methods and adjusting the time of follow-up from 14 days for COVID-19 to 21 days for EVD. COVID-19 tracing of asymptomatic individuals on the seventh and 12th days can also be combined with testing for Ebola to maximize epidemiological surveillance.^12^

In light of previous EVD experience, contact tracing has been organized at local and regional levels during the COVID-19 pandemic. The same framework applies to the current EVD outbreak. Therefore, the response to the Ebola virus includes mobile laboratories to target affected communities. Nowadays, the response to COVID-19 has developed to create many community laboratories for point-of-care (e.g., GeneXpert) COVID-19 testing.^13^

Additionally, due to the significance of engaging communities and response rate during the Ebola virus epidemic in the Guinea Republic, the process to obtain constant response from different communities was highly prioritized from the inception of the response to COVID-19.

## LESSONS LEARNED FROM COVID-19 AND THEIR EFFECT ON THE MANAGEMENT OF THE EBOLA OUTBREAK

Experience obtained during the fight against SARS-CoV-2 in the Republic of Guinea can be used against EVD. The three principal lessons include continuation of vital health services, revitalizing healthcare, and protecting healthcare workers.^14^ For example, public health authorities in Guinea have organized door-to-door awareness campaigns and home visits to prevent or provide early diagnosis for malaria.^15^

Developing similar campaigns for noninfectious diseases, such as hypertension and diabetes mellitus, should be a high priority in the face of EVD. This is particularly important in light of recent evidence suggesting that effective management of metabolic syndrome amid the COVID-19 pandemic can decrease hospitalizations by 20%.^16^

The need to protect healthcare workers is a bitter lesson from June 2020, when SARS-CoV-2 infected 160 of the republic’s 2,000 healthcare workers. Every healthcare worker is vital for the continuation of care in Guinea. With more infectious strains of SARS-CoV-2 arising, along with the Ebola virus, there is great need to ensure that medical professionals are well-equipped with protective equipment.

## CHALLENGES AND PRIORITIES FOR EBOLA RESPONSE AMID THE COVID-19 PANDEMIC

The main priorities to manage Ebola during the COVID-19 pandemic are outlined in this section. Identifying and addressing these challenges requires joint effort and coordination between the authorities and international health institutions.

1. **Overwhelmed healthcare system:** According to the WHO, the Republic of Guinea faces financial dysfunction, as well as a shortage of healthcare workers and infrastructure.^17,18^ Therefore, the national healthcare system can be easily overwhelmed by the concomitant rise in COVID-19 and Ebola cases. Optimizing transportation of medical equipment and personnel is a priority to maximize the efficacy of the available resources. At the same time, it is crucial to increase the per capita health expenditure, which currently accounts to just $9 per year.^19^ This increase is necessary to increase the number of hospital beds and healthcare workforce available per 10,000 population.^20^ Currently, the medical workforce density accounts to less than 1.5 per 10,000 population.^21^ Creating platforms of training related to prevention and hygiene and providing free Iinternet access to the public who wish to access them, can decrease the number of hospitalizations securing beds for patients with COVID-19 and EVD.
2. **Low COVID-19 and Ebola Testing:** The need to establish an Ebola tracing infrastructure has a negative impact on the capacity of COVID-19 testing. State laboratories dedicate all their resources to reverse-transcriptase polymerase chain reaction for COVID-19 and Ebola testing. In this frame, testing for other diseases or conditions becomes almost impossible, and detection of COVID-19 and SARS-CoV-2 is slower than is should be. At this rate, morbidity and mortality associated not only with COVID-19 and EVD but also with other conditions has increased. Hence, it is a priority to assign COVID-19 and Ebola referral laboratories per region. It is also important to provide these laboratories with separate financial resources and, if possible, equipment for these diseases to maintain a fair level of functionality for other conditions’ diagnostics and monitoring.
3. **Misinformation:** Misinformation through mainstream and social media is highly prevalent in the Republic of Guinea, resulting in a rise in COVID-19 and Ebola virus cases. Evidence from the 2013 previous outbreak has also shown that misinformation leading to distrust between the community and biomedics has led to decreased hospital admissions;^22^ if 10% more admissions would have occurred, the previous Ebola outbreak could have been reduced by 26%.^23^ Therefore, it is high time to put more effort in community COVID-19 and EVD awareness. Community and tribal leaders, along with local and national celebrities, should be targeted as multipliers of credible information.
4. **Accessibility to vaccines:** Vaccines are necessary to build immunity to COVID-19 and EVD. Accessibility to vaccines in remote areas of the Guinea Republic is low because of the treacherous terrain and the lack of appropriate storing facilities.^24^ With the aforementioned shortages in personnel, it is a priority to involve nongovernmental organizations such as the International Medical Corps in vaccine distribution and delivery. However, any active infection spread in local communities can decrease the rate of vaccinations. Hence, to maximize the number of people who can receive vaccines, it is vital to provide those living in rural areas with a good water supply and adequate hygiene and sanitation.^25^

## RECOMMENDATIONS

To address the aforementioned challenges and priorities, the following recommendations should be taken into consideration:

1. Contact tracing: identify contacts of Ebola cases to reduce further spread.
2. Equip healthcare infrastructure with adequate facilities to treat and isolate people infected with Ebola.
3. Establish guidelines for funeral practices for patients who die from or are suspected of having Ebola.
4. Emphasize scientific communication at the community level.
5. Supply outstanding healthcare equipment to minimize the spread of Ebola and its effects on the public health system.

In addition, awareness and engaging communities is important. Direct communication to dwellers in the communities where Ebola affects is a necessity. Further, people in the affected communities must comply with restrictive measures and guidelines and understand how Ebola threatens their own health. In this way, misinformation can be addressed.

The following recommendations can be implemented in a long-term strategy to increase accessibility to vaccines and alleviate the overwhelmed healthcare system.

1. Develop efficient information and research units to conduct epidemiological analysis by mapping, identification, and tracing the cases. WHO experts can contribute, and the international academic community can also be involved in developing innovative approaches; however, local scientists need to have a leading role because of their proximity to the particular circumstances in the country.
2. Implement efficient leadership and government involvement in financing the health system. Mobilizing sufficient state funds to strengthen the infrastructure and prioritizing appropriate resource allocation in cooperation with experts from the WHO, United Nations, and UNICEF.
3. Improve service delivery by developing healthcare facilities, constructing effective transport networks, and making them accessible to those who cannot afford them.

**Figure 1.**
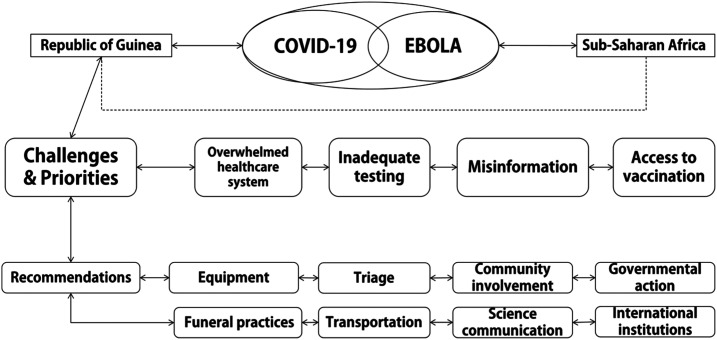
An overview of challenges, priorities, and recommendations for the containment of Ebola virus amid the COVID-19 pandemic.

An overview of challenges, priorities, and recommendations is provided in Figure 1.

## CONCLUSION

As the Guinea Republic strives to reduce the spread of COVID-19, it is important to consider past and current situations—in particular, experiences from the epidemic of Ebola. However, the country needs to use current facilities and guidelines of COVID-19 while addressing communities’ concerns to involve them in the strategic response plan to avoid the double burden of COVID-19 and EDV. The Guinea Republic must increase funding to its weak healthcare facilities and management to maintain health equity and stability and public health security for the country’s people. It is hoped that the Guinea Republic response control to reduce the effect of COVID-19 will be successful through proper screening, testing, behavior of the people, and trace contact of affected and suspected communities; this will be possible if there are sufficient local, regional, and national amenities, facilities, and healthcare professionals. Overall, the Guinea Republic has a better chance to effectively contain COVID-19 if the challenges stemming from the EVD outbreak are addressed in a rigorous and timely fashion.
